# Considering Psychosocial Factors When Investigating Blood Pressure in Patients with Short Sleep Duration: A Propensity Score Matched Analysis

**DOI:** 10.1155/2021/7028942

**Published:** 2021-11-30

**Authors:** Ningjing Qian, Dandan Yang, Huajun Li, Siyin Ding, Xia Yu, Qingqiu Fan, Zhebin Yu, Shenfeng Ye, Hualiang Yu, Yaping Wang, Xiaohong Pan

**Affiliations:** ^1^Department of Cardiology, The Second Affiliated Hospital, Zhejiang University School of Medicine, Hangzhou, China; ^2^Institute of Environmental Medicine, Karolinska Institutet, Stockholm, Sweden; ^3^Department of Psychiatry, The Second Affiliated Hospital, Zhejiang University School of Medicine, Hangzhou, China

## Abstract

Few studies have considered psychosocial characteristics when investigating the associations between sleep duration and blood pressure (BP). In this study, we took propensity score matching (PSM) to adjust for psychosocial characteristics when comparing BP between individuals with short sleep duration and those with normal sleep duration. A total of 429 participants were included. 72 participants with sleep duration ≤6 h and 65 participants with sleep duration >6 h were matched after PSM. We compared office BP, 24-hour BP, and prevalence of hypertension in the populations before and after PSM, respectively. In the unmatched population, participants with sleep duration ≤6 h were observed with higher office diastolic BP (DBP) and 24-h systolic BP (SBP)/DBP (all *P* < 0.05). In the matched populations, the differences between the two groups (sleep duration ≤6 h vs. sleep duration >6 h) in office DBP (88.4 ± 10.9 vs. 82.5 ± 11.1 mm Hg; *P*=0.002), 24-h SBP (134.7 ± 12.0 vs. 129.3 ± 11.6 mm Hg; *P*=0.009), and 24-h DBP (83.4 ± 9.9 vs. 78.1 ± 10.1 mm Hg; *P*=0.002) become more significant. Participants with sleep duration ≤6 h only show higher prevalence of hypertension based on 24-h BP data, while analysis after PSM further revealed that these with sleep duration ≤6 h presented about 20% higher prevalence of elevated BP up to office diagnosed hypertension threshold. Therefore, psychosocial characteristics accompanied with short sleep duration should be fully valued in individuals at risks for elevated BP. This trial is registered with NCT03866226.

## 1. Introduction

There is growing interest in the impact of psychosocial and lifestyle factors on cardiovascular diseases [1–3]. Hypertension, one of the most common cardiovascular diseases, is a serious clinical and public health issue. The number of adults living with hypertension doubled over the past 30 years globally [4]. With high prevalence but low awareness, treatment, and control rates, hypertension not only largely contributes to the risks of cardiovascular morbidity and mortality but also lays a substantial economic and health burden on the society. Despite the advances in the detection, evaluation, and management, the control and prognosis of hypertension remain poor [2–7]. Notably, raised blood pressure (BP) levels are associated with a broad range of modifiable risk factors, such as smoking, alcohol, diabetes, increased body weight, and insomnia, given that major international guidelines have attracted great importance to the psychosocial and lifestyle modification in the management of hypertension [1, 3, 7].

The association between sleep and hypertension has been investigated in several dimensions, including sleep duration, sleep quality, and insomnia symptoms such as difficulty maintaining sleep [8, 9]. Objective short sleep duration is the most biologically severe phenotype of insomnia [10], and it has been the most commonly used indicator in studies to examine the relationship [11, 12]. However, a link of sleep duration insufficiency with elevated BP risk was observed in earlier studies [13, 14]; more recent surveys seem instead to weakly support a statistically significant association, which makes the relationship more “ambiguous” [15–17]. Moreover, previous studies have not taken psychosocial characteristics, such as anxiety, depression, and stress, into consideration which differ between people with normal sleep duration and those with short sleep duration [12, 18, 19]. These divergent characteristics may also contribute to the raised BP levels and the risk of hypertension [2, 3, 7]. The missing of these data raises concerns regarding the potential for the distortion of the true relationship between sleep duration and hypertension—the psychosocial characteristics weakening or strengthening the impact of sleep duration on BP.

In this study, we investigate the office and 24-hour ambulatory BP levels and prevalence of hypertension among individuals with short sleep duration compared with individuals with normal sleep duration in a population-based cross-sectional study. Propensity score matching (PSM) was performed to adjust for psychosocial and other conventional risk factors.

## 2. Methods

### 2.1. Study Population and Patients' Eligibility

**Figure 1:**
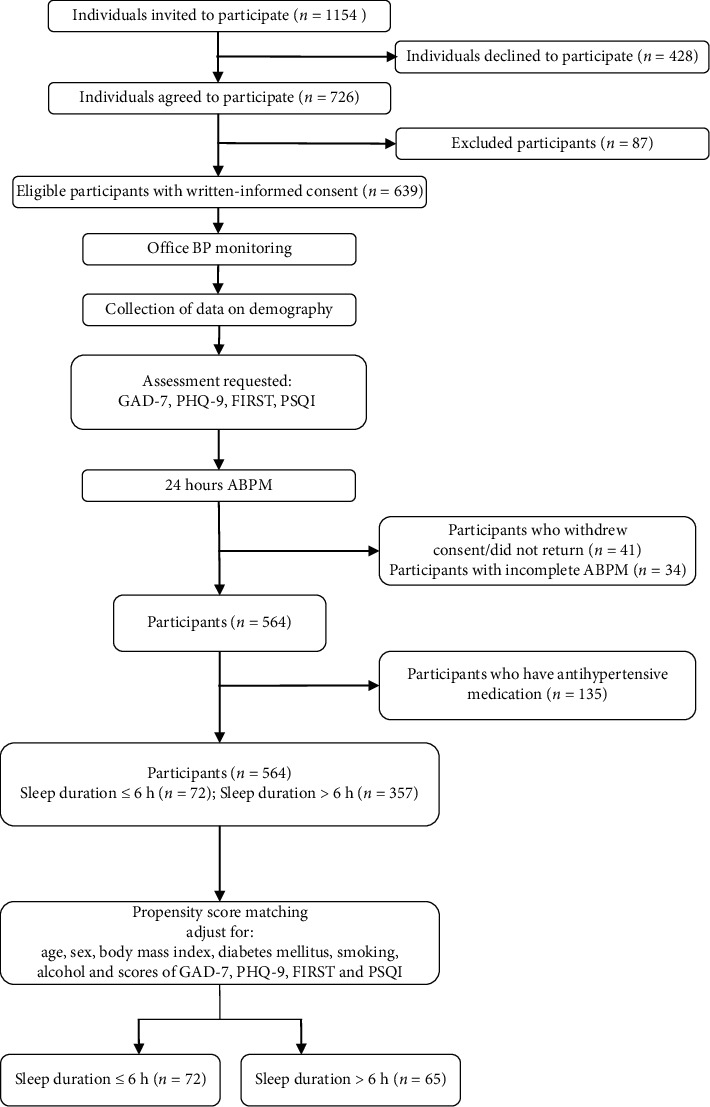
The selection flowchart for the study participants. ABPM indicates ambulatory blood pressure monitoring; BP, blood pressure; GAD-7, Generalized Anxiety Disorder Scale-7; PHQ-9, Patient Health Questionnaire-9; FIRST, Ford Insomnia Response to Stress Test; PSQI, Pittsburgh Sleep Quality Index.

This study was based on a cross-sectional survey conducted at the Second Affiliated Hospital of Zhejiang University School of Medicine between March and November 2019. 564 outpatients between ages of 18 and 80 years, with no limitation of gender, who completed ambulatory blood pressure monitoring (ABPM) in routine work day, were enrolled. The main exclusion criteria included (i) history of diagnosed insomnia, currently under treatment; (ii) history of diagnosed obstructive sleep apnea; (iii) history of other diagnosed sleep disorders, including narcolepsy/hypersomnias, circadian rhythm sleep disorder, parasomnias, and sleep-related dyskinesia; (iv) history of diagnosed psychosis, including schizophrenia, bipolar disorder, depression, anxiety, obsessive-compulsive disorder, phobia, somatoform disorder, and stress-related disorders; (v) heart failure, with New York Heart Association Class III-IV; (vi) atrial fibrillation and frequent atrial or ventricular extrasystoles; (vii) ongoing alcohol or drug abuse; (viii) use of sedative hypnotic medication within 1 week; (ix) pregnancy; (x) ongoing night shift work; (xi) ongoing need of taking care of families at home during night within 1 week; (xii) incomprehensible or unwilling to fill in informed consent or questionnaire. All the clinical history was collected by trained physicians, and the diagnoses of diseases were according to the International Classification of Diseases (ICD) 10. Individuals with a history of diagnosed insomnia or diagnosed obstructive sleep apnea, other diagnosed sleep disorders, ongoing night shift work, and ongoing need of taking care of families at home during night within 1 week were excluded because they could likely have sleep circadian rhythm disturbance, which might cover up or amplify the association between sleep duration and BP. Individuals with a history of diagnosed psychosis were excluded since they might be poor at cooperation with ABPM and self-evaluation. Special populations who are recommended to take ABPM with caution, such as patients with atrial fibrillation, pregnancy, were excluded as well [5]. In addition, 135 individuals who reported with antihypertensive medication were excluded from the further analysis.  Figure 1 illustrates the selection flowchart for the study participants.

### 2.2. Office Blood Pressure Monitoring

Office BP was automatically measured following the suggested steps in the office setting at the beginning of the visit. In brief, participants were seated for 5 min in a quiet room before the first measurement. With the appropriate cuff, measurements were repeated 3 times, 1-2 min apart by Omron HBP1300 monitor (Omron Healthcare Co. Ltd., Kyoto, Japan). Basic office BP was based upon the means of 3 readings.

### 2.3. Ambulatory Blood Pressure Monitoring

ABPM devices including Oscar 2 monitor (Suntech Medical, USA), Mobil-O-Graph (I.E.M. GmbH, Germany) and Beneware Model ABP-021 (Beneware, China) were all calibrated within 1 month and randomly selected. Participants were equipped with ABPM devices in the nondominant arm at the outpatient clinic, and they underwent ABPM out of office during the 24-h period [1, 5]. The monitor was set with cuff inflations every 20 minutes during 6 : 00 to 22 : 00 and every 30 minutes during 22 : 00 to 6 : 00. The criteria for considering an ABPM complete are at least 70% validity of planned readings [5]. All participants were provided with instruction on ABPM procedures as recommended in guidelines. 24-h BP was defined as average of readings over 24 h. ABPM thresholds for defining hypertension includes 24-h systolic BP (SBP)/diastolic BP (DBP) ≥130/80 mm Hg [2, 3, 7].

### 2.4. Assessment of the Impact of ABPM on Sleep

The impact of ABPM on sleep was assessed after ABPM by a visual analogue scale [20] from 0 to 10 with 0 representing “free” and 10 indicating “unable to fall asleep.”

### 2.5. Sleep Assessment

The investigation of sleep duration was based on the answer to the question “During the past month, how many hours of actual sleep did you get at night?” in the Pittsburgh Sleep Quality Index (PSQI). PSQI is a self-report questionnaire comprising seven “components” designed by Buysse et al. The seven “components,” including sleep duration, sleep quality, sleep disruption, sleep latency, sleep efficiency, daytime disturbance, and use of sleep medications, can be analyzed independently or yield a global score to provide a global subjective sleep evaluation over the past month. The higher scores on PSQI represent poorer global sleep conditions [21]. The Chinese version of PSQI has been widely used in Chinese population with good reliability and validity [22]. Sleep duration ≤6 hours and >6 hours were defined as short sleep duration and normal sleep duration [23].

### 2.6. Anxiety Assessment

Symptoms of anxiety were assessed by the generalized anxiety disorder-7 (GAD-7). GAD-7 is a reliable and valid tool for identifying generalized anxiety by self-report in the general population with score range from 0–21. The higher scores on GAD-7 represent higher severity levels of anxiety symptom [24]. The Chinese version of GAD-7 has been validated and applied with satisfactory [25].

### 2.7. Depression Assessment

Symptoms of depression were assessed by the Patient Health Questionnaire-9 (PHQ-9). PHQ-9 is a self-report questionnaire widely used to measure the severity of depression. The score of PHQ-9 ranges from 0–27 and higher scores indicates higher severity levels of depression [26]. The Chinese version of PHQ-9 has been widely used with good reliability and validity [25, 27].

### 2.8. Sleep Reactivity Assessment

Sleep reactivity, that is, the preexisting sleep vulnerability response to stress, was evaluated by the Ford Insomnia Response to Stress Test (FIRST). FIRST is developed as a self-report measure with score ranging from 9 to 36, and higher scores predict higher sleep reactivity [28, 29]. The Chinese version of FIRST has been validated and considered as a good screening tool for sleep reactivity [30].

### 2.9. Propensity Score Matching

PSM was performed to balance the possible bias and differences in psychosocial characteristics between individuals with short sleep duration and individuals with normal sleep duration. The propensity score was estimated for each individual using a logistic regression model including the following variables: age, sex, body mass index (BMI), diabetes mellitus, smoking, alcohol and scores of GAD-7, PHQ-9, FIRST, and PSQI. 1 : 1 nearest neighbor matching method was performed with a caliper width of 0.1 standard deviation of the propensity score and without replacement.

### 2.10. Statistical Analysis

All statistical analysis was done with SPSS version 24.0 (IBM Corporation, Armonk, NY, USA). Continuous variables, including age, SBP/DBP, heart rate, scores of GAD-7, PHQ-9, and FIRST were expressed as mean (standard deviation, SD) after checking that data did not deviate significantly from a normal distribution and categorical variables were described as number (%) of subjects. Differences in variables between groups were analyzed with chi-square test for categorical variables and independent sample *t*-test for continuous variables. The statistically significant level was set at *P* < 0.05.

## 3. Institutional Review Board Approval

The ethics approval of the study was obtained from the Ethics Committee of the Second Affiliated Hospital of Zhejiang University School of Medicine. All eligible individuals included had provided written informed consent.

## 4. Results

A total of 564 participants who underwent ABPM between March and November 2019 were included, of which 135 with antihypertensive medication were excluded. And, a total of 429 participants (72 participants with sleep duration ≤6 h and 357 participants with sleep duration >6 h) were included in the analysis of this study (Figure 1). Participants with sleep duration ≤6 h and those with sleep duration >6 h were matched 1 : 1 by PSM. 72 participants with sleep duration ≤6 h and 65 participants with sleep duration >6 h were compared, and the demographic and psychosocial characteristics were well balanced (Table 1). In the unmatched sample, the GAD-7 score in participants with sleep duration ≤6 h was 3.7 ± 4.0 compared to 3.2 ± 3.5 in participants with sleep duration >6 h. The PHQ-9 score was 3.8 ± 3.6 for participants with sleep duration ≤6 h vs. 2.9 ± 3.3 for participants with sleep duration >6 h. And, the FIRST score was 14.7 ± 6.1 in participants with sleep duration ≤6 h compared to 14.1 ± 4.9 in participants with sleep duration >6 h. After PSM, no significant differences were observed between the groups (Table 1). The impact of ABPM on sleep was also similar between the groups after PSM, which excluded its potential influence on BP levels (Supplemental Table 1).

The BP values obtained by office measurement and ABPM measurement were compared between groups, and the results before and after PSM are shown in Table 2. Before PSM, significant differences in a number of BP indices were observed. Participants with sleep duration ≤6 h had higher office DBP (88.4 ± 10.9 vs. 84.7 ± 12.1 mm Hg; *P*=0.015), 24-h SBP (134.7 ± 12.0 vs. 130.7 ± 13.6 mm Hg; *P*=0.023), and 24-h DBP (83.4 ± 9.9 vs. 79.9 ± 11.0 mm Hg; *P*=0.013), compared with those with sleep duration >6 h. The differences between the two groups (sleep duration ≤6 h vs. sleep duration >6 h) in office DBP (88.4 ± 10.9 vs. 82.5 ± 11.1 mm Hg; *P*=0.002), 24-h SBP (134.7 ± 12.0 vs. 129.3 ± 11.6 mm Hg; *P*=0.009), and 24-h DBP (83.4 ± 9.9 vs. 78.1 ± 10.1 mm Hg; *P*=0.002) become more significant after PSM.

The prevalence of elevated BP according to office and ABPM thresholds for diagnosing hypertension were further compared as shown in Table 3. Before PSM, the group with sleep duration ≤6 h performed higher prevalence of elevated office DBP (48.6% vs. 33.3%; *P*=0.014), 24-h DBP (68.1% vs.49.9%; *P*=0.005), and 24-h SBP/DBP (75.0% vs. 61.6%; *P*=0.031). And, consistent with the results of BP values, the group with sleep duration > 6 h was with more significantly lower prevalence of elevated office DBP, 24-h DBP, and 24-h SBP/DBP after PSM. A significant difference in the prevalence of diagnosed hypertension on ABPM data between the groups was observed both before and after PSM. Meanwhile, analysis after PSM further revealed that participants with sleep duration ≤6 h presented about 20% higher prevalence of elevated BP up to office diagnosed hypertension threshold.

## 5. Discussion

This study is the first propensity-matched comparison of individuals with short sleep duration and individuals with normal sleep duration to investigate the association between sleep duration and BP. The present study confirms that individuals with short sleep duration have higher BP levels and higher prevalence of hypertension than those with normal sleep duration. And, in the selected PSM populations adjusted for psychosocial and conventional risk characteristics, the differences in BP levels and prevalence of hypertension between the two groups turn to be more statistically significant. Our findings indicate that short sleep duration is significantly associated with elevated BP and psychosocial characteristics accompanied with short sleep duration should be fully valued and be potential targets for preventing and managing hypertension.

To the best of our knowledge, few studies have adjusted for psychosocial characteristics when examining the associations between sleep duration and BP. Sleep duration insufficiency usually doesn't occur alone, and for many patients suffering from sleep duration insufficiency, psychosocial problems, such as anxiety, depression, and stress, prevail simultaneously [18, 19, 31]. Thus, to investigate the relationship between sleep duration and BP should consider these factors and adjust for the bias. However, it is impracticable and costly of a randomized control trial to cope with this question. In this study, PSM was performed to matching individuals suffering from short sleep duration to individuals having normal sleep duration with similar baseline characteristics and psychosocial status, which overcame the limitation. We found that, in the unmatched populations, higher BP levels in all BP indices and increased prevalence of hypertension were detected in individuals with short sleep duration (≤6 h). Nevertheless, there was no significant difference in prevalence of hypertension between individuals with short sleep duration (≤6 h) and those with normal sleep duration (>6 h), and short sleepers were only observed with higher BP levels in office DBP, 24-h DBP, and nighttime DBP after PSM. The results in the unmatched populations are consistent with previous studies [32–34]. The study by Kalmbach et al. demonstrates that short sleepers have higher prevalence rates of hypertension compared to those with normal sleep duration [34], and the prospective study in Taiwan by Deng et al. suggests that short sleepers are found with 8% higher risk for elevated BP (the thresholds defined as office SBP ≥130 mm Hg or DBP  ≥85 mm Hg) during the following from 1996 to 2014 [32]. It has remained unclear whether short sleep duration alone could truly account for elevated BP since these previous studies have not controlled for anxiety, depression, or other psychosocial factors. The present study optimizes the comparison between groups by PSM. Taken together, these findings suggest individuals with short sleep duration are likely to have higher BP levels while the relationship between sleep duration and BP could not be interpreted uniformly across individuals. Rather, the impact of short sleep duration on BP levels could be differential across populations due to myriad factors, such as anxiety, depression, and stress.

The most important implication of this study is that, for short sleepers or insomniacs with hypertension and at the risks of hypertension, sleep should not be the only focus of modification, psychosocial status values as well. It has been reported that individuals with frequent insomnia are more likely have poor hypertension control. Compared with individuals without insomnia, the risk of poor hypertension control can be increased by 23% in men with insomnia and 18% in women with insomnia, respectively [35]. Emotional sufferings such as anxiety, depression, and stress are frequent in patients with insomnia or sleep insufficiency [19]. And, it has been known that anxiety, depression, and stress have positive association with hypertension, increasing the risk of hypertension and making the intervention against hypertension more difficult [36–39]. These emotional sufferings and sleep insufficiency may further lead a “multiplier” effect to hypertension. Strategies dealing with emotional problems, for example, cognitive-behavioural treatment for insomnia (CBT-I), have attracted great importance to facilitate insomnia treatment and shown improved outcomes with good efficacy [18, 19]. Therefore, taking a dual approach by targeting at sleep and psychosocial status should be recommended, in particular, for short sleepers or insomniacs with hypertension and at the risks of hypertension, controlling hypertension at fairly low cost and high efficiency.

There are a few limitations of our study. First, our study is a cross-sectional study so that our findings fail to provide causal relationship between sleep duration and hypertension nor the impact of longitudinal sleep changes on BP and related cardiovascular disease risks. Second, since our study is a single-center study, all the participants enrolled came from the same region. We have not taken covariates such as dietary habit and race into analysis, and the sample size is limited. Thus, the findings cannot represent the overall population, and further studies are needed to extrapolate the findings to other populations. Third, only 24-h SBP/DBP of ABPM results were used to compare out-of-office BP between normal sleepers and short sleepers. Although previous studies have shown high agreement in daytime and nighttime BP results of ABPM between predefined time and self-report approach in general population [40], daytime and nighttime BP results analyzed according to the predefined time were not used since our comparison was conducted between short sleepers and normal sleepers. The differences between the predefined sleep period and the real sleep periods could influence the analyzed BP results, which may further affect the accuracy of comparison. Finally, sleep duration was self-report evaluated in this study rather than objectively by actigraphy or polysomnography. We have to admit that objective sleep assessment could provide more detailed information, and the validity of self-report sleep duration might be limited. However, actigraphy or polysomnography is not practical in outpatient populations, but self-report is much more acceptable and convenient. On the other hand, some studies demonstrate that self-report approach could be more sensitive than device detecting approach [41, 42].

In summary, our study suggests individuals with short sleep duration are likely to have higher BP levels as well as higher prevalence of hypertension, even more significant after adjusted for psychosocial characteristics. The results of our study emphasize that not only sleep duration, but also psychosocial status should be paid attention to in the study of risk for elevated BP and in the management of hypertension. These modifiable factors could be effective approach to prevent hypertension and improve the efficacy of antihypertensive treatment.

## Figures and Tables

**Table 1:** Baseline clinical characteristics of the unmatched and matched populations.

	Unmatched population (*n* = 429)		Propensity score matched 1 : 1 (*n* = 137)		
	Sleep duration ≤6 h	Sleep duration >6 h	Sleep duration ≤6 h	Sleep duration >6 h	*P*
No. of patients	72	357	72	65	
Age, mean (±SD)	47.7 (12.9)	44.9 (14.2)	47.7 (12.9)	47.1 (13.9)	0.797
Male, % *(n)*	58.3 (42)	209 (58.5)	58.3 (42)	64.6 (42)	0.451
BMI (kg/m^2^), mean (±SD)	24.0 (3.0)	24.0 (3.3)	24.0 (3.0)	24.1 (2.8)	0.787
Diabetes mellitus, % *(n)*	4.2 (3)	12 (3.4)	4.2 (3)	7.7 (5)	0.380
Alcohol, % *(n)*	30.6 (22)	85 (23.8)	30.6 (22)	32.3 (21)	0.825
Smoking, % *(n)*	23.6 (17)	69 (19.3)	23.6 (17)	24.6 (16)	0.891
GAD-7, mean (±SD)	3.7 (4.0)	3.2 (3.5)	3.7 (4.0)	3.5 (3.4)	0.801
PHQ-9, mean (±SD)	3.8 (3.6)	2.9 (3.3)	3.8 (3.6)	3.1 (3.0)	0.237
FIRST, mean (±SD)	14.7 (6.1)	14.1 (4.9)	14.7 (6.1)	14.2 (4.8)	0.579

SD indicates standard deviation; BMI, body mass index; GAD-7, Generalized Anxiety Disorder Scale-7; PHQ-9, Patient Health Questionnaire-9; FIRST, Ford Insomnia Response to Stress Test.

**Table 2:** Blood pressure values of the unmatched and matched populations.

	Unmatched population (*n* = 429)			Propensity score matched 1 : 1 (*n* = 137)		
	Sleep duration ≤6 h	Sleep duration >6 h	*P*	Sleep duration ≤ 6 h	Sleep duration >6 h	*P*
No. of patients	72	357		72	65	
Office SBP, mean (±SD)	139.4 (14.2)	137.0 (17.0)	0.270	139.4 (14.2)	136.2 (16.4)	0.230
Office DBP, mean (±SD)	88.4 (10.9)	84.7 (12.1)	**0.015**	88.4 (10.9)	82.5 (11.1)	**0.002**
Office HR, mean (±SD)	78.4 (9.7)	78.4 (11.5)	0.980	78.4 (9.7)	77.7 (10.2)	0.663
24-h SBP, mean (±SD)	134.7 (12.0)	130.7 (13.6)	**0.023**	134.7 (12.0)	129.3 (11.6)	**0.009**
24-h DBP, mean (±SD)	83.4 (9.9)	79.9 (11.0)	**0.013**	83.4 (9.9)	78.1 (10.1)	**0.002**
24-h HR, mean (±SD)	75.7 (7.1)	73.8 (8.8)	0.095	75.7 (7.1)	74.2 (7.5)	0.248

SD indicates standard deviation; BP indicates blood pressure; SBP, systolic blood pressure; DBP, diastolic blood pressure; HR, heart rate.

**Table 3:** Prevalence of elevated blood pressure in the unmatched and matched populations.

	Unmatched population (*n* = 429)			Propensity score matched 1 : 1 (*n* = 137)		
	Sleep duration ≤6 h	Sleep duration >6 h	*P*	Sleep duration ≤6 h	Sleep duration >6 h	*P*
No. of patients	72	357		72	65	
Office BP (mm hg)						
SBP ≥140, % *(n)*	41.7 (30)	44.0 (157)	0.718	41.7 (30)	35.4 (23)	0.451
DBP ≥90, % *(n)*	48.6 (35)	33.3 (119)	**0.014**	48.6 (35)	21.5 (14)	**0.001**
24-h BP (mm hg)						
SBP ≥130, % *(n)*	55.6 (40)	53.2 (190)	0.717	55.6 (40)	46.2 (30)	0.272
DBP ≥80, % *(n)*	68.1 (49)	49.9 (178)	**0.005**	68.1 (49)	44.1 (28)	**0.003**
Office SBP/DBP≥ 140 and/or ≥ 90 (mm hg), % *(n)*	58.3 (42)	51.8 (185)	0.313	58.3 (42)	41.5 (27)	**0.050**
24-h SBP/DBP ≥130 and/or ≥ 80 (mm hg), % *(n)*	75.0 (54)	61.6 (220)	**0.031**	75.0 (54)	53.8 (35)	**0.010**

BP indicates blood pressure; SBP, systolic blood pressure; DBP, diastolic blood pressure.

## Data Availability

The data that support the findings of this study are available from the corresponding author upon reasonable request.

## Supplementary Materials

Supplementary Materials Supplemental table 1 shows the effect of ABPM on sleep in the unmatched and matched populations.
