# Supplementary material for: Considering Psychosocial Factors When Investigating Blood Pressure in Patients with Short Sleep Duration: A Propensity Score Matched Analysis
Source: Int J Hypertens. 2021 Nov 30;2021:7028942. doi: 10.1155/2021/7028942 (PMC8651353; doi:10.1155/2021/7028942)
Supplement: Supplementary Materials — Supplemental table 1 shows the effect of ABPM on sleep in the unmatched and matched populations. [file 7028942.f1.docx]

| Supplemental table 1 The effect of ABPM on sleep in the unmatched and matched populations. | | | | | | |
| --- | --- | --- | --- | --- | --- | --- |
|  | Unmatched-Population  (n = 429) | | | Propensity score matched 1:1  (n = 137) | | |
|  | Sleep duration ≤ 6h | Sleep duration > 6h | *P* | Sleep duration ≤ 6h | Sleep duration > 6h | *P* |
| No. patients | 72 | 357 |  | 72 | 65 |  |
|  | 3.49 (3.24) | 3.75 (2.96) | 0.501 | 3.49 (3.24) | 4.08 (3.02) | 0.273 |
| ABPM indicates, ambulatory blood pressure monitoring. | | | | | | |
